# Prevalence and Phase Variable Expression Status of Two Autotransporters, NalP and MspA, in Carriage and Disease Isolates of *Neisseria meningitidis*

**DOI:** 10.1371/journal.pone.0069746

**Published:** 2013-07-25

**Authors:** Neil J. Oldfield, Suzan Matar, Fadil A. Bidmos, Mohammed Alamro, Keith R. Neal, David P. J. Turner, Christopher D. Bayliss, Dlawer A. A. Ala’Aldeen

**Affiliations:** 1 Molecular Bacteriology and Immunology Group, School of Molecular Medical Sciences, University of Nottingham, Nottingham, United Kingdom; 2 Department of Biological Sciences and Medical Analysis, University of Jordan, Amman, Jordan; 3 Department of Genetics, University of Leicester, Leicester, United Kingdom; 4 School of Community Health Sciences, University of Nottingham, Nottingham, United Kingdom; Beijing Institute of Microbiology and Epidemiology, China

## Abstract

*Neisseria meningitidis* is a human nasopharyngeal commensal capable of causing life-threatening septicemia and meningitis. Many meningococcal surface structures, including the autotransporter proteins NalP and MspA, are subject to phase variation (PV) due to the presence of homopolymeric tracts within their coding sequences. The functions of MspA are unknown. NalP proteolytically cleaves several surface-located virulence factors including the 4CMenB antigen NhbA. Therefore, NalP is a phase-variable regulator of the meningococcal outer membrane and secretome whose expression may reduce isolate susceptibility to 4CMenB-induced immune responses. To improve our understanding of the contributions of MspA and NalP to meningococcal-host interactions, their distribution and phase-variable expression status was studied in epidemiologically relevant samples, including 127 carriage and 514 invasive isolates representative of multiple clonal complexes and serogroups. Prevalence estimates of >98% and >88% were obtained for *mspA* and *nalP*, respectively, with no significant differences in their frequencies in disease versus carriage isolates. 16% of serogroup B (MenB) invasive isolates, predominately from clonal complexes ST-269 and ST-461, lacked *nalP.* Deletion of *nalP* often resulted from recombination events between flanking repetitive elements. PolyC tract lengths ranged from 6–15 bp in *nalP* and 6–14 bp in *mspA*. In an examination of PV status, 58.8% of carriage, and 40.1% of invasive *nalP*-positive MenB isolates were *nalP* phase ON. The frequency of this phenotype was not significantly different in serogroup Y (MenY) carriage strains, but was significantly higher in invasive MenY strains (86.3%; *p*<0.0001). Approximately 90% of MenB carriage and invasive isolates were *mspA* phase ON; significantly more than MenY carriage (32.7%) or invasive (13.7%) isolates. This differential expression resulted from different mode *mspA* tract lengths between the serogroups. Our data indicates a differential requirement for NalP and MspA expression in MenB and MenY strains and is a step towards understanding the contributions of phase-variable loci to meningococcal biology.

## Introduction

The encapsulated diplococcus, *Neisseria meningitidis,* persists in the upper respiratory tract of 10–30% of individuals without causing clinical symptoms [1], [2]. In rare cases, hyper-virulent meningococci invade the epithelial layers and enter the bloodstream resulting in rapidly fatal septicemia and meningitis [1]. The use of conjugate polysaccharide vaccines targeting some disease-associated serogroups has helped limit the impact of disease in several countries; however, a prerequisite for comprehensive prevention in the developed world will be an effective and widely used vaccine against serogroup B meningococci (MenB) [3]. One vaccine developed for this purpose is 4CMenB (Bexsero). This has been licensed for use in European countries and contains four antigenic components: factor H binding protein (fHbp), Neisserial adhesin A (NadA), Neisseria heparin binding antigen (NhbA) and outer membrane vesicles from a New Zealand epidemic strain [4]. This vaccine is predicted to provide protection against ∼78% of European invasive MenB strains suggesting a requirement for additional vaccine components [5].

Meningococci elaborate numerous cell-surface and secreted virulence factors which facilitate colonization of, persistence in, and damage to, the host [6]. One important class are the autotransporter (or type V secreted) proteins. Eight have been identified in meningococci: IgA1 protease, NhhA, AutA, AutB, NadA, App, NalP (also known as AspA) and MspA (also known as AusI) [7], five of which were first discovered in our laboratory [8]–[11]. Autotransporters share common structural features including a conserved C-terminal β-barrel translocator domain and a more variably conserved functional N-terminal passenger domain. The translocator inserts in the outer membrane and is required for passenger domain translocation [12], [13]. Following export, the passenger domain of some autotransporters undergoes proteolytic cleavage, resulting in the release of biologically active fragments from their respective cell-bound translocator domain [12]. The functions of autotransporters vary widely, but often relate to virulence and include acting as enzymes, adhesins or cytotoxins [14].

The coding sequences of two meningococcal autotransporters, NalP and MspA, contain poly-cytosine tracts which differ in length between strains suggesting that their expression is phase variable [10], [11], [15], [16]. Phase variation (PV) is the reversible switching ON or OFF of protein expression, often mediated by slipped-strand mispairing during DNA replication at repeat tracts [17]. PV may allow bacteria to adapt to changes in their microenvironment [18] and evade adaptive immune responses [19]. The precise functions of MspA are unknown, although a role in adhesion of meningococci to host cells has been suggested [11]. Transcriptome analysis using an *ex vivo* model of human whole blood infection revealed significant increases in *mspA* expression in blood supporting an important role for MspA during invasive disease [20].

MspA and NalP both exhibit auto-proteolytic activity resulting in the release of passenger domain fragments into the external milieu. The pattern of secreted MspA fragments varies extensively between strains and is also influenced by the activity of NalP [11], [15]. In addition to targeting itself and MspA, NalP proteolytically processes other surface proteins including App and IgA1 protease [10], [16], LbpB (lactoferrin binding protein B) [21] and the 4CMenB antigen NhbA [22]. The consequences of the proteolytic activity of NalP on meningococcal pathogenesis are yet to be fully determined, but a *nalP* knockout mutant was more sensitive to killing by human whole blood compared to the wild-type, suggesting that NalP is a factor (directly or indirectly) involved in the survival of the meningococcus in this niche [20]. Furthermore, recent work has shown the impact of NalP activity on DNA-dependent biofilm formation. In meningococci not expressing NalP, the DNA binding fragments of NhbA and IgA1 protease are retained on the cell surface resulting in efficient biofilm formation. In NalP-expressing meningococci, NalP-mediated cleavage and release of these DNA-binding fragments leads to reduced biofilm formation [23]. NalP expression may also reduce isolate susceptibility to 4CMenB-induced immune responses since this vaccine contains the NalP target NhbA; albeit *in vitro* bactericidal assays suggest that NhbA processing by NalP does not significantly affect the susceptibility of isolates to killing by polyclonal anti-NhbA antibody [22].

In summary, NalP and MspA are outer membrane/secreted proteins with important roles in meningococcal virulence. However, understanding the contributions these autotransporters make to meningococcal-host interactions is hampered by a lack of detailed studies on their distribution and phase-variable expression status in epidemiologically relevant samples. In this study, we determined the presence and expression status of both *nalP* and *mspA* in two recent isolate collections. This analysis showed that both genes are found at high frequency in both carriage and invasive isolates, and revealed mechanisms which lead to loss of *nalP* in *nalP­*-negative strains. Tract length analysis revealed differences in the proportions of strains in ON/OFF expression states between carriage and invasive isolate collections, and between serogroups, indicating differential requirements for MspA and NalP expression in different niches and in different meningococcal lineages.

## Materials and Methods

### Isolate collections

A subset of 127 isolates, taken from a larger strain collection obtained from a cohort follow up study of meningococcal carriage in students at Nottingham University, UK during 2008–09, was used in this study. Briefly, a cohort of 190 students was sampled for meningococcal nasopharyngeal carriage at four time-points throughout the academic year [24]. One representative colony per individual per time point was sub-cultured for the production of glycerol stocks and DNA extracts using the DNeasy Blood and Tissue kit (Qiagen). Strains were confirmed as meningococci using molecular methods and subsequently PorA, FetA and multilocus sequence typed and PCR-serogrouped [24]. A total of 214 isolates were obtained, but for persistently carried meningococcal strains (*i.e.* strains with identical fine-typing characteristics carried by the same individual over multiple time-points), only the first isolate was included in this study to yield a core set of 127 isolates comprising all 89 strains obtained at the first time-point and 38 additional strains from other time-points representing acquisition or clonal replacement events [24]. The Meningitis Research Foundation Meningococcus Genome Library database (containing the unfinished genome sequences of all disease isolates for the epidemiological year 2010–11 in England, Wales and Northern Ireland) was interrogated by BLAST (last analyzed April 2013) at http://pubmlst.org/perl/bigsdb/bigsdb.pl?db=pubmlst_neisseria_mrfgenomes. Additional DNA and protein sequence analyses were carried out using DNAMAN v4.13 (Lynnon BioSoft).

### PCR amplification and DNA sequence analysis

*nalP* was detected using primers nalPF1 and nalPR1 (Table 1). For some strains the resulting amplicons were sequenced using nalPF1, nalPR1 and nalPR2 (Table 1). *nalP* deletion and replacement events were characterized by amplifying using NMB1968F and NMB1970R (Table 1) and sequencing the amplified products using the same primers and internal primers. Detection of *mspA* was undertaken using mspAF1 and mspAR1 (Table 1); these products were sequenced using these primers and mspAR2 (Table 1). PolyC tract lengths in *nalP* and *mspA* were determined by a combination of DNA sequencing and sizing of PCR fragments. For the latter, fragments were amplified using a 6-carboxyfluorescein (FAM)-labeled primer (nalPF2-FAM or mspAF2-FAM, respectively) and a non-labeled primer (nalPR2 or mspAR2, respectively) in a 10 µl PCR reaction. PCR products were A-tailed by the addition of a 4 µl reaction mix containing 0.8 µl PCR buffer (5×), 0.4 µl 2mM dATP, 0.04 µl Taq and 2.76 µl distilled H_2_O, followed by incubation at 72°C for 45 min. 0.5 µl volumes of PCR products (1∶100 diluted in H_2_O) were mixed with Hi-Di Formamide and GeneScan 500 LIZ size standard (Applied Biosystems), followed by electrophoresis on an ABI 3130 Genetic Analyzer (Applied Biosystems). GeneScan data were analyzed using Peak Scanner v1.0 software (Applied Biosystems) using the local southern size calling method.

**Table 1:** Primers used in this study.

Primer	DNA sequence
nalPF1	GTTGCAACAACACTTTCTGCCTGC
nalPR1	GCAGGTTGTCGTTGCTCATCCACG
nalPR2	CAGGCGCTTCCTTCCGCATATACG
NMB1968F	CACGGTTTGGAAGAATATCTGC
NMB1970R	TGCAAACAACACCGAATGCAGC
mspAF1	TATCGGCAACAACAGGCAACAGGC
mspAR1	TTTTGGGCACGCGTCAGAATGCCG
mspAR2	GGTAGGCACCCAAATCGGCATAGC
nalPF2-FAM	AAATGTGCAAAGACAGAAGCATGC
mspAF2-FAM	GCAGTCCGCATGGAAGCAGACAGC

### Phenotypic analysis

Expression of NalP and MspA in representative strains was confirmed by immunoblot analysis of concentrated secreted protein preparations. Secreted proteins were prepared from 200 ml cultures of meningococci grown at 37°C overnight in Dulbecco's Modified Eagle Medium (DMEM; Invitrogen). Meningococci were harvested by centrifugation (8,000× *g* for 10 min) and supernatants filtered (Steritop 0.22 µm pore size; Millipore) and concentrated approximately 200-fold (Vivaspin-20 protein concentrator, 30-kDa molecular weight cut-off; Sartorius). Secreted proteins were electrophoretically separated using 10% polyacrylamide mini-gels (Mini-Protean III, Bio-Rad) and transferred to nitrocellulose membranes. Membranes were blocked with 1% [w/v] bovine serum albumin (BSA), 0.1% [v/v] Tween®20 in 1× phosphate buffered saline [PBS] overnight at 4°C. Membranes were probed with rabbit anti-MspA [11] or rabbit anti-NalP [10] diluted 1∶4000 or 1∶500, respectively in blocking solution and incubated for 2 h. After washing three times in 1× PBS with 0.1% Tween^®^20 (PBST), membranes were incubated for 2 h with goat anti-rabbit IgG-alkaline phosphatase conjugate (Sigma), at a dilution of 1∶30,000 in blocking solution. After washing with PBST, the blots were developed using BCIP/NBT-Blue liquid substrate (Sigma).

### Statistical analyses

Statistical analyses were carried out using GraphPad Prism version 5. *P* values were derived with a two-tailed Fisher's exact test.

## Results

### Distribution of *mspA* and *nalP* in carriage and disease isolates

We first examined by PCR the distribution of both genes in a collection of recent UK carriage isolates (*n* = 127) containing representatives from eight different serogroups (B, C, E, H, W, X, Y and Z), in addition to non-serogroupable strains [24]. We observed that 99.2% of carriage isolates harbored *mspA*, whilst 92.9% had *nalP* (Table 2). For comparison, *in silico* examination of the genome sequences of 514 recent UK invasive isolates revealed that 97.9% of isolates harbored *mspA*, whilst 87.7% had *nalP* (Table 2). To exclude the possibility that the apparent lack of *mspA* or *nalP* in some genome sequences was due to a lack of coverage, BLAST analysis was undertaken using the sequences of genes flanking *mspA* and *nalP* (in *N. meningitidis* MC58, NMB1997 and NMB1999 for *mspA* and NMB1968 and NMB1970 for *nalP* ¸ respectively). Highly similar sequences could be detected in all 514 genome sequences, confirming that the 11 (*mspA*) and 63 (*nalP*) invasive isolates identified were negative for the gene of interest (Tables S1 and S2, respectively). No strains in either collection lacked both genes and there was no significant difference in the frequency of *mspA* or *nalP* in disease versus carriage isolates (Table 2). Overall, 98.1% of strains examined possessed *mspA* (629/641) and 88.8% (569/641) possessed *nalP*.

**Table 2:** Distribution of *mspA* and *nalP* in carriage (*n* = 127) and disease (*n* = 514) isolates.

Gene	Strain collection	No. of strains containing gene	No. of strains lacking gene	*P* value
*mspA*	carriage	126 (99.2%)	1 (0.8%)	0.4763
	invasive	503 (97.9%)	11 (2.1%)	
*nalP*	carriage	118 (92.9%)	9 (7.1%)	0.1164
	invasive	451 (87.7%)	63 (12.3%)	

### Deletion mechanisms at the *nalP* locus

**Figure 1:**
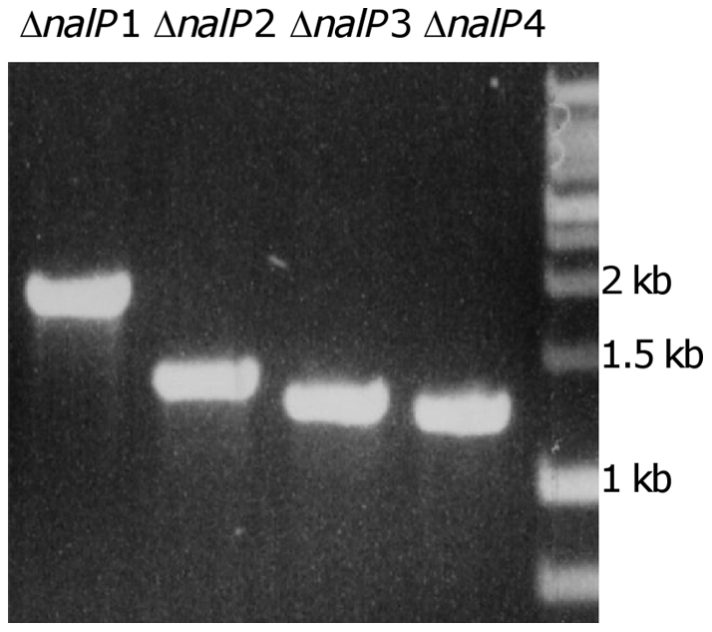
Variations in the size of the *nalP* deletion locus amongst *N. meningitidis* carriage isolates. The *nalP* deletion loci were amplified with primers specific for flanking genes. Amplicons were grouped into four classes based on size; representative samples are shown. Δ*nalP*1 (V128; Dec), Δ*nalP*2 (V130; Nov), Δ*nalP*3 (V199; Nov) and Δ*nalP*4 (V206; Dec).

Meningococcal isolates lacking *mspA* have been described previously [11], [15]. In contrast, to our knowledge, this is the first report describing meningococcal strains lacking *nalP*. We therefore investigated *nalP* deletion mechanisms by amplification of the deletion loci from the nine *nalP-*negative carriage isolates using primers specific for the adjacent genes (NMB1968 encoding aldehyde dehydrogenase A and NMB1970 encoding a putative para-aminobenzoate synthase). This analysis revealed that the PCR products generated from *nalP*-negative carriage strains ranged in size from *ca.* 2-kb to *ca.* 1.2-kb and could be separated into four groups (designated Δ*nalP*1, Δ*nalP*2, Δ*nalP*3 and Δ*nalP*4) based on amplicon size (Fig. 1), with the following numbers of isolates in each group: Δ*nalP*1, 1; Δ*nalP*2, 3; Δ*nalP*3, 2 and Δ*nalP*4, 2 (Table 3). No amplicon could be obtained from the remaining *nalP-*negative strain perhaps indicating sequence polymorphisms at the annealing sites of one (or both) of the primers used.

**Table 3:** Carriage isolates which lacked *nalP*.

Volunteer	Time point^a^	Strain typing data*^b^*	*nalP* deletion group*^c^*
V128	Dec	NG:P1.5,2–12:ND:ST823 (cc198)	Δ*nalP*1
V64	May	NG:P1.17,9:F1-82:ST823 (cc198)	ND
V118	Nov	E:P1.19,15:ND:ND	Δ*nalP*2
V130	Nov	NG:P.1.7–2,13–2:ND:ST2591 (cc461)	Δ*nalP*2
V218	Nov	E:P1.19,15:F5-28:ND	Δ*nalP*2
V199	Nov	B:P.1.22,9:F5-5:ST283 (cc269)	Δ*nalP*3
V207	Nov	B:P.1.19–1,15-11:F1-7:ST269 (cc269)	Δ*nalP*3
V192	Dec	NG:P1.18–1,3:F3-6:ST3808 (cc254)	Δ*nalP*4
V206	Dec	E:P1.5–1,10–8:F3-6:ST254 (cc254)	Δ*nalP*4

a Time points indicate when each strain was isolated from a particular carrier. Carriers were sampled in November and December 2008, and February and May 2009.

b, c ND, not determined.

**Figure 2:**
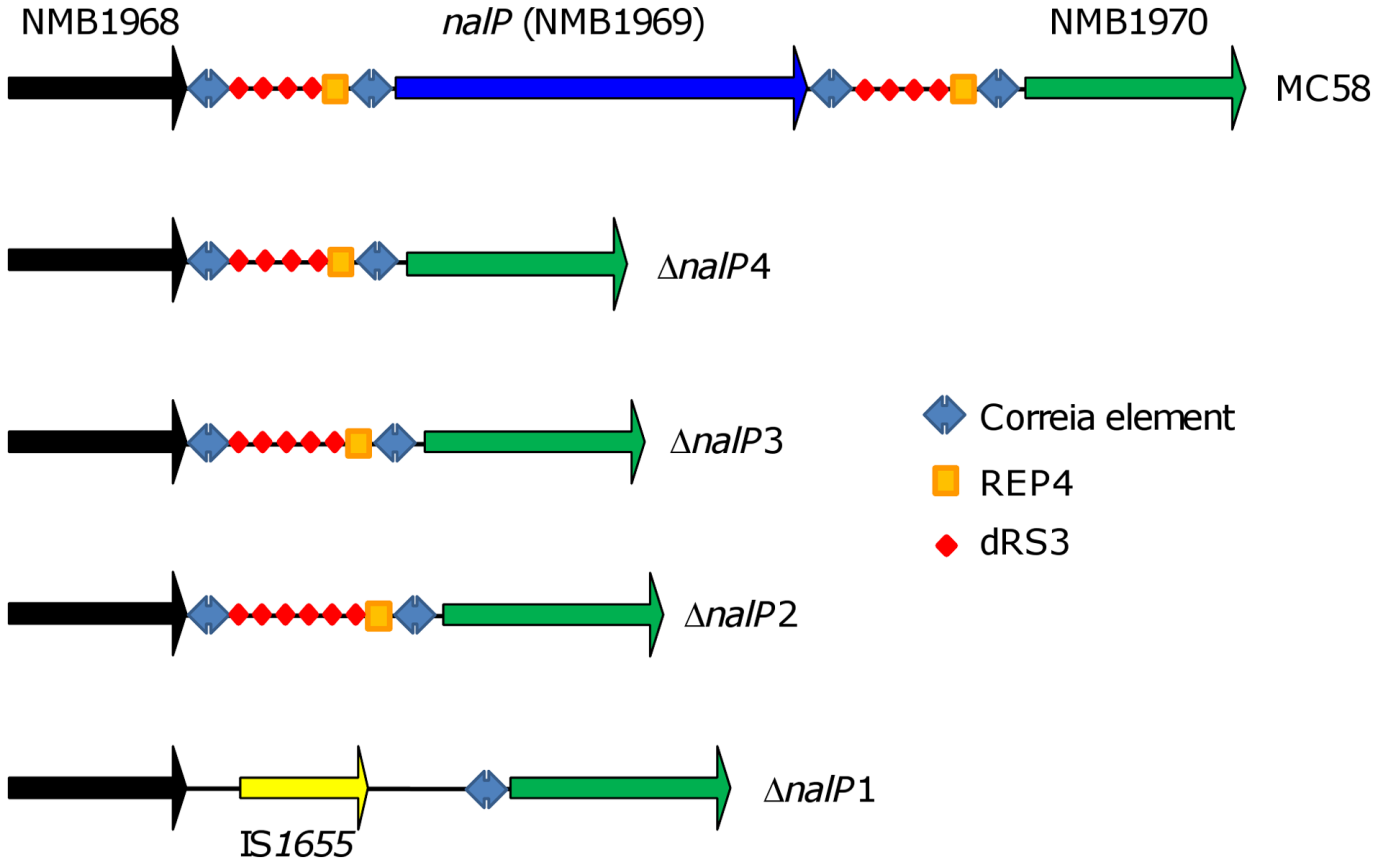
Genetic arrangement of the *nalP* locus in *N. meningitidis* isolates. Unidirectional arrows represent ORFs. Repetitive elements are represented by different symbols. dRS3 consensus sequence: ATTCCCNNNNNNNNGGGAAT; REP4 consensus sequence: AAGACCGTCGGGCATCTGCAGCCGTC. Other repetitive sequences are not shown for clarity. Note that the figure is not drawn to scale but is a representation of the various repeat elements and genes present at the *nalP* locus in *N. meningitidis* MC58 and four *nalP*-negative carriage isolates representing Δ*nalP*1, Δ*nalP*2, Δ*nalP*3 and Δ*nalP*4 classes.

To identify the deletion mechanisms responsible for this variation in amplicon size, the DNA sequences of the PCR products shown in Fig. 1 were generated (GenBank accession numbers KF207543-KF207546) and compared to the *nalP* locus of the MenB strain MC58 [25]. In MC58, *nalP* is flanked by multiple repetitive DNA sequences including two clusters of four dRS3 elements (one cluster upstream and one cluster downstream of *nalP*). Each dRS3 cluster is flanked by a downstream 26-bp REP4 sequence and upstream and downstream Correia elements (Fig. 2). Sequencing of *nalP* deletion loci revealed a variable number of dRS3 repeats in three of the four deletion types (Δ*nalP*2, Δ*nalP*3 and Δ*nalP*4; Fig. 2) suggesting that deletion of *nalP* had occurred via different recombination events between repetitive sequences in regions upstream and downstream of *nalP*, leaving residual fragments of varying lengths between the flanking genes (Fig. 2). In the case of the Δ*nalP*1-class, the sequenced fragment contained an ORF with 100% identity to the IS*1655* transposase gene indicating that dRS3-independent *nalP* deletion events also occur. Further *in silico* analysis revealed that multiple isolates harboring Δ*nalP*2 and Δ*nalP*3 class deletions could be identified within the 63 *nalP­-*negative invasive isolates, thus confirming that similar *nalP* deletion types are present in both invasive and carriage isolates (Table S2).

### Distribution of *nalP* deletion mechanisms within clonal complexes (CCs)

Strikingly, phylogenetic analyses of strains revealed that the majority of *nalP-*negative isolates were restricted to a few CCs, primarily the ST-461 complex (all 11 strains lacked *nalP*) and the ST-269 complex (where 47% of 104 isolates lacked *nalP*) (Table 4). Notably, the latter is a hyper-invasive lineage, with a disease/carriage ratio of 2.8 [2]. Additionally, it was also apparent that the ST-461 and ST-269 CCs were associated with a different *nalP* deletion class, with the former associated with the Δ*nalP*2-class, whilst strains from the latter complex commonly harbored Δ*nalP*3-class deletions (Tables 3 and S2). Other CCs also harbored apparently high proportions of strains lacking *nalP,* for example 67% and 100% of strains from CCs ST-198 and ST-254, respectively, lacked *nalP*, however examination of larger numbers of strains from these CCs will be required to determine whether *nalP* is commonly absent in these lineages.

**Table 4:** Phylogenetic breakdowns of both carriage and invasive strain collections showing the frequency of *nalP* deletions within clonal complexes.

Clonal Complex	Carriage only		Invasive only		Carriage & Invasive	
	Number of isolates	Number of isolates lacking *nalP*	Number of isolates	Number of isolates lacking *nalP*	Number of isolates	Number of isolates lacking *nalP* (%)^a^
Not assigned	25	2	46	1	71	3 (4)
174	17	0	9	0	26	0
23	15	0	57	0	72	0
1157	12	0	3	0	15	0
60	12	0	15	0	27	0
167	10	0	4	0	14	0
213	6	0	37	0	43	0
22	6	0	13	0	19	0
103	5	0	5	1	10	1 (10)
**269**	**4**	**2**	**100**	**47**	**104**	**49 (47)**
198	3	2	0	0	3	2 (67)
41/44	3	0	136	3	139	3 (2)
53	3	0	0	0	3	0
254	2	2	0	0	2	2 (100)
32	2	0	29	0	31	0
35	1	0	8	1	9	1 (11)
**461**	**1**	**1**	**10**	**10**	**11**	**11 (100)**
11	0	0	21	0	21	0
Others	0	0	21	0	21	0
Total	127	9	514	63	641	72

a (%) indicates the percentage of isolates within each clonal complex which were *nalP­*-negative.

### PV status of *mspA* and *nalP* in carriage and invasive strains

**Figure 3:**
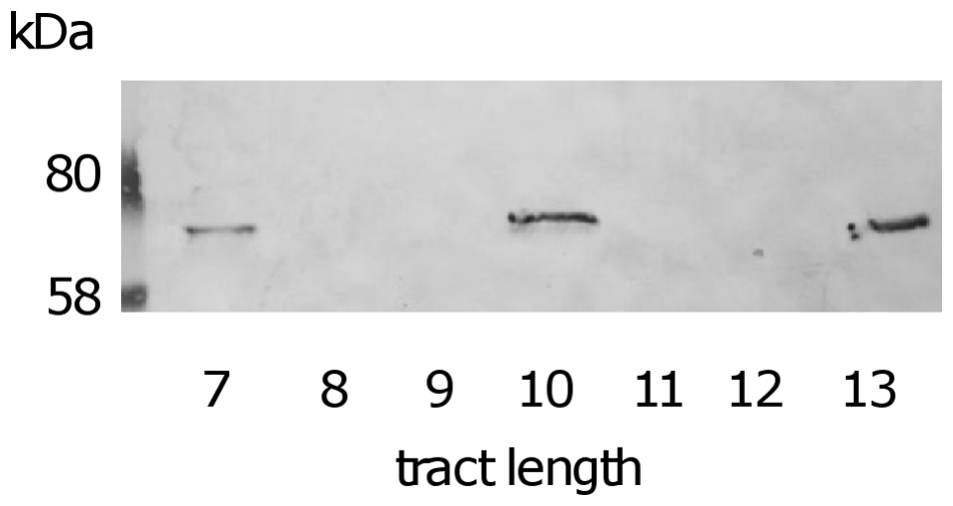
Immunoblot confirmation that *nalP* tract lengths of 7, 10 or 13Cs results in NalP expression. Secreted protein preparations from representative strains were probed with rabbit anti-NalP antisera to confirm that only *nalP* homopolymeric tract lengths of 7, 10 or 13Cs are consistent with the gene being in-frame and phase ON. Secreted NalP fragments ranged in size between *ca.* 68–70kDa due to sequence polymorphisms between alleles [10], [16]. Strains used (tract length in parentheses): V199; May (7), V1114; Nov (8), V78; Nov (9), V131; Nov (10), V182; Nov (11), V169; Dec (12) and V193; Nov (13).

Having shown that most meningococcal isolates contain *mspA* and *nalP*, we examined the ON/OFF status of both autotransporters in the carriage isolates by a combination of sizing of fluorescent PCR products (*i.e.* GeneScan) and DNA sequencing. The expression status of NalP in representative carriage strains with different tract lengths was confirmed by immunoblot analysis of secreted protein preparations using specific antisera indicating that the genetic screen for ON/OFF PV states correlated with the phenotypic expression of NalP (Fig. 3). Similar analysis confirmed that a homopolymeric tract length of 6, 9 or 12Cs resulted in MspA expression (data not shown).

The PV status of *mspA* and *nalP* in invasive strains was also determined by *in silico* examination of their genome sequences. Tract lengths could not be defined for a minority of strains in this collection since in these strains the *mspA* or *nalP* polyC tract spanned multiple contigs. However, from 503 *mspA*-positive invasive strains, we were able to determine the *mspA* tract length in 500 isolates (99.4%), and for 451 *nalP*-positive invasive strains, we determined the *nalP* tract length in 430 isolates (95.3%).

**Figure 4:**
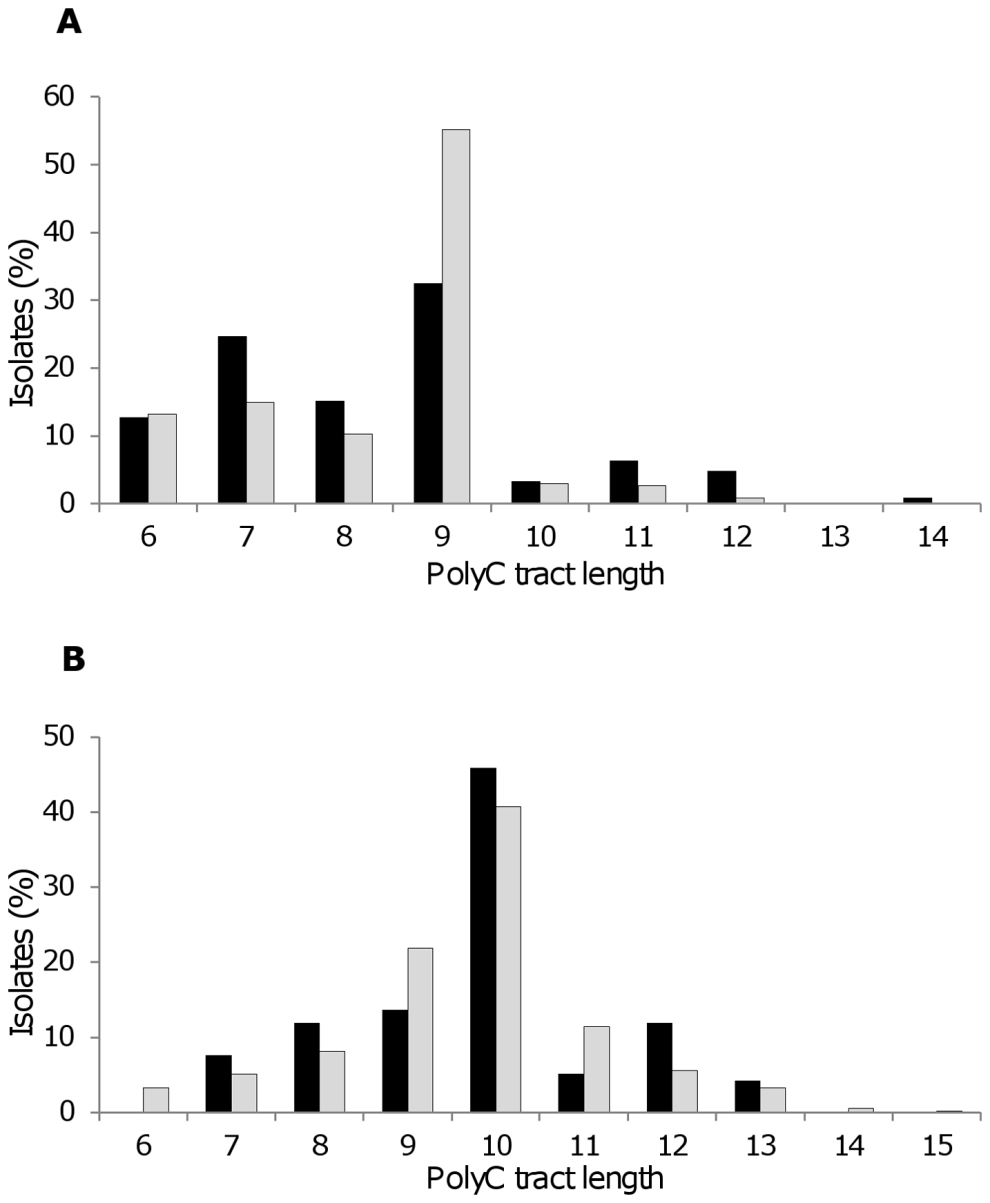
Distribution of tract lengths for *mspA* (A) and *nalP* (B) in invasive and carriage isolates. Invasive isolates shown are a sub-set of the 2010–11 UK isolates available in the Meningitis Research Foundation Meningococcus Genome Library database. Carriage isolates were from a study of meningococcal carriage in students at Nottingham University, UK during 2008–09. Black bars, carriage; grey bars, invasive. An ON PV state is produced by 6, 9 or 12Cs for *mspA* and by 7, 10 or 13Cs for *nalP*.

Tract lengths ranged from 6–14 bp (mode = 9, phase ON) in *mspA* (Fig. 4A), and 6–15 bp (mode = 10; phase ON) in *nalP* (Fig. 4B). There was no significant difference (*p* = 0.1188) in the frequency of *nalP* phase ON (tract length of 7, 10 or 13Cs) strains in disease versus carriage isolates (Table 5); approximately 50% of strains in both collections were *nalP* phase ON. In contrast, a highly significant difference (*p*<0.0001) was detected in the frequency of *mspA* phase ON (tract length of 6, 9 or 12Cs) strains in the carriage versus invasive collections (Table 6), with *mspA* being significantly more often ON in invasive isolates compared to carriage isolates (69.2% compared to 50.0%).

**Table 5:** Distribution of phase ON and OFF *nalP* genes in carriage and disease isolates by serogroup.

Serogroup	Carriage		Invasive		*P* value
	No. of strains phase ON	No. of strains phase OFF	No. of strains phase ON	No. of strains phase OFF	
B	10 (58.8%)^a^	7 (41.2%)	125 (40.1%)^b^	187 (59.9%)	0.1366
Y	36 (69.2%)^a^	16 (30.8%)	63 (86.3%)^b^	10 (13.7%)	0.0259
Others	22 (44.9%)	27 (55.1%)	23 (51.1%)	22 (48.9%)	0.6797
Total	68 (57.6%)	50 (42.4%)	211 (49.1%)	219 (50.9%)	0.1188

a No statistically significant difference in the frequency of the *nalP* phase ON phenotype between MenB and MenY carriage isolates (*p = *0.5547).

b Statistically significant difference in the frequency of the *nalP* phase ON phenotype between MenB and MenY invasive isolates (*p<*0.0001).

**Table 6:** Distribution of phase ON and OFF *mspA* genes in carriage and disease isolates by serogroup.

Serogroup	Carriage		Invasive		*P* value
	No. of strains phase ON	No. of strains phase OFF	No. of strains phase ON	No. of strains phase OFF	
B	17 (89.5%)^a^	2 (10.5%)	328 (86.1%)^b^	53 (13.9%)	1.0000
Y	17 (32.7% )^a^	35 (67.3%)	10 (13.7%)^b^	63 (86.3%)	0.0150
Others	29 (52.7%)	26 (47.3%)	8 (17.4%)	38 (82.6%)^c^	0.0004
Total	63 (50.0%)	63 (50.0%)	346 (69.2%)	154 (30.8%)	<0.0001

a Statistically significant difference in the frequency of the *mspA* phase ON phenotype between MenB and MenY carriage isolates (*p<*0.0001).

b Statistically significant difference in the frequency of the *mspA* phase ON phenotype between MenB and MenY invasive isolates (*p<*0.0001).

c Includes 15 strains from ST-11 which harbor a mutation in *mspA* interrupting the gene. All 15 strains were phase OFF at the polyC tract.

### Influence of serogroup on PV status

The invasive strain collection used in this study had the following composition: 76.3% MenB, 14.4% MenY and 9.3% from other serogroups. In contrast, the carriage isolate collection comprised 15% MenB, 40.9% MenY and 44.1% from other serogroups. To understand whether these differences were influencing our observations regarding shifts in ON/OFF status between carriage and invasive collections, we examined tract lengths in both isolate collections by serogroup, with particular focus on MenB and MenY strains.

For *nalP* harbored by MenY strains, there was a significant difference between the frequency of the phase ON phenotype during carriage and invasive disease (69.2% ON in carriage to 86.3% ON in invasive; *p = *0.0259). However, no corresponding statistically significant changes were apparent for MenB strains and the remaining non-B, non-Y strain group (Table 5 and Fig. S1) leading to no overall significant change in the proportions of strains in ON or OFF PV states in carriage versus invasive collections. In each serogroup and under each condition (carriage or invasive), the mode tract length in *nalP* was 10Cs (phase ON). Notably, the frequency of the ON phenotype was significantly higher in invasive MenY strains than invasive MenB strains (86.3% versus 40.1%; *p<*0.0001), whilst the frequency of the ON phenotype was not statistically different (*p = *0.5547) between carried MenY and MenB strains.

For *mspA,* serogroup-specific differences were more evident (Table 6 and Fig. S2). For MenB strains, 89.5% of carriage, and 86.1% of invasive isolates, respectively, were *mspA* phase ON. In contrast, only 32.7% of carriage and 13.7% of invasive MenY strains were *mspA* phase ON, with the shift to an *mspA* OFF phenotype in MenY invasive strains being statistically significant (*p = *0.015). For both carriage and invasive isolates, the frequency of the *mspA* phase ON phenotype was significantly higher in MenB strains than MenY strains (*p<*0.0001 for both comparisons). The basis for this difference was apparent from our tract length analysis which showed that the mode tract length for MenB strains was 9Cs (phase ON), whilst for MenY strains the mode was 7Cs (phase OFF) (Fig. S2). The breakdown by serogroup also clarified the apparent overall switch to *mspA* being phase ON during invasive disease. This was likely to be a consequence of the overrepresentation of MenB strains (with *mspA* ON in 86.1% of them) in the invasive collection (*n = *328 MenB phase ON strains from 500 invasive strains examined; 65.6%) compared to their frequency in the carriage collection (*n = *17 MenB phase ON strains from 126 carriage strains examined; 13.5%). Taken together, this data identifies correlations between serogroup and specific tract lengths, and hence differential expression states, of MspA in MenB and MenY meningococci.

### Prevalence of a premature TAG stop codon in *mspA*

Previous studies on *mspA* have noted that in some strains from ST-11, the gene contains a single nucleotide mutation (C to T) which places a premature TAG stop codon in frame [11], [15]. Thus in these strains the gene is interrupted even if the number of Cs in the *mspA* polyC tract would lead to an in-frame ORF. Our collection of carriage strains contained no strains from this lineage; however 21 invasive isolates belonged to ST-11 (Table 4). *In silico* analysis showed that 15 of these harbored the C to T mutation, but all contained polyC tracts containing a number of nucleotides consistent with the gene being phase OFF. This analysis also confirmed that this mutation was limited to ST-11 strains, since no other strains were identified that harbored this C to T substitution.

## Discussion

NalP and MspA are meningococcal autotransporter proteases, but their precise biological significance remains unclear [7]. In addition to functional studies, investigating the distribution and PV-mediated expression of *mspA* and *nalP* in disease and carriage isolates may lead to an improved understanding of the role of these proteins in pathogenesis and disease progression. Previous studies have looked at the prevalence of *mspA* and *nalP* in small numbers of isolates (typically <20 strains) [10], [11], [15], [16]. In this study, we investigated the prevalence of both genes in a considerably larger (*n* = 641) collection of epidemiologically relevant strains. Overall, 98.1% of strains examined possessed *mspA* and 88.8% possessed *nalP* with no significant differences between the frequencies of either gene in carriage versus invasive isolates. This is in contrast to the phase variably expressed gene, *hmbR,* (encoding for the HmbR haemoglobin receptor) which has been detected at a significantly higher frequency in disease compared to carriage isolates [26], [27].

The identification of *nalP-*negative strains was especially significant since, to our knowledge, strains lacking *nalP* have not been noted previously. Characterization of the deletion loci in carriage isolates identified four types of deletion event involving either recombination events between highly similar flanking sequences or replacement with IS*1655.* Similar events have previously been associated with the deletion of meningococcal genes encoding other surface localized proteins including FetA, PorA and HpuAB [27]–[30]. Multiple strains from the invasive collection could also be assigned by BLAST to one of the deletion classes. Unfortunately, the remaining *nalP* deletion loci spanned multiple contigs, and their complex and repeat-rich nature precluded *in silico* reassembly. Completion of the genome sequences in the MRF library database will allow further strains to be assigned to each of four deletion classes and may allow for the identification of additional mechanisms of *nalP* deletion.

The *nalP* gene was absent in 72 of the strains examined, 83% (60/72) of these deletion events were associated with two lineages, ST-461 and ST-269. The latter is a diverse lineage composed of over 30 distinct STs, which can be split into two distinct groups, the ST-269 and ST-275 clusters, respectively [31]. All the ST-269 *nalP-*negative strains were from STs found in the ST-269 cluster (data not shown). Interestingly, the ST-461 and ST-269 lineages were each associated with a specific type of *nalP* deletion event. Stable lineages of this type suggest that *nalP* deletion is infrequent, possibly because the selective advantage associated with deletion of the gene is low. However, it should be noted that other CCs, for example, the ST-41/44 complex, contained strains lacking *nalP* at low frequencies suggesting that sporadic *nalP* deletion events can also occur.

Having shown that most meningococcal isolates contain *mspA* and *nalP*, we examined the polyC tract lengths of both autotransporters in both isolate collections. PolyC tract lengths in *mspA* ranging from 6 to 10 bp long have been described [15]. Our data extends this to show that tract lengths up to 14 bp long can be harbored by meningococci. Similarly, tract lengths in *nalP* ranging in size between 9–15 bp have previously been noted [10]; our analysis extends this to show that smaller tract lengths (down to 6 bp) can also be found.

Importantly, our tract length analysis revealed some important differences regarding PV-mediated expression of the two autotransporters between serogroups and in different niches. For MenB strains, the frequency of NalP expression was not significantly different between carriage or invasive strain collections, with expression (phase ON) predicted in 58.8% and 40.1% of carriage and invasive strains, respectively. However, the former estimate was based on only 17 strains; analysis of a larger number of carried MenB isolates may lead to a refinement in this estimate. Intriguingly, our estimates for NalP expression were considerably higher in MenY strains, indicating a greater requirement for NalP expression in MenY lineages, particularly during invasive disease. It has previously been shown that a MenB *nalP* knockout mutant was more sensitive to killing by human whole blood compared to the wild-type. This suggests that NalP is a factor involved in blood survival (in MenB at least) [20]. The importance of this protective role in blood is undermined by our finding that 16% (61/392) of the MenB disease isolates from England, Wales and Northern Ireland in 2010–11 lacked *nalP* and furthermore, that *ca.* 60% of *nalP-*positive invasive MenB strains were not expressing the protein. This would suggest that either NalP is not essential to the development of invasive disease or that another protein may compensate for the loss of NalP in MenB. It is feasible that in other serogroups (such as MenY), the greater frequency of NalP expression during invasive disease is due to the absence of this compensating protein, however this requires further experimental confirmation.

One target for NalP-mediated cleavage is NhbA [22]. A recombinant derivative of this 60-kDa lipoprotein is one of four antigenic components in the newly licensed anti-MenB vaccine, 4CMenB (Bexsero) [32]. Outer membrane-localized NhbA can be incompletely cleaved by NalP to release a 22-kDa fragment, corresponding to the C-terminal region of NhbA, into the external milieu [22]. This has led to concerns that NalP expression may reduce the susceptibility of MenB isolates to 4CMenB-induced immune responses. Our findings suggest that this will not be a universal effect as *nalP* is absent in some MenB lineages, whilst a high proportion (59%; 194/329) of *nalP*-positive MenB strains examined were not expressing this protein.

NalP proteolytic activity has also been shown to influence DNA-dependent biofilm formation, with maximal biofilm formation seen in meningococcal strains not expressing NalP [23]. The phase variable expression of NalP has therefore been suggested to influence meningococcal dispersal and the colonization of new niches and carriers [23]. Although biofilm formation is likely to a complex and multi-factorial process, our data would suggest that since MenB strains are generally less likely to be expressing NalP, they would be more likely to efficiently form biofilms than MenY strains. The influence of NalP on biofilm formation in MenY strains and any resulting effects on their transmissibility and propensity to cause disease compared to MenB strains remain unclear.

For *mspA*, niche-specific and serogroup-specific differences in PV-expression were also apparent. Approximately 90% of *mspA*-positive carriage and invasive MenB strains were expressing MspA. This complements previously reported data which showed that *mspA* expression in MenB significantly increases in blood [20] and suggests an important role for MspA during the development of MenB invasive disease (and also potentially during carriage). In contrast, estimates of MspA expression in *mspA-*positive MenY strains were significantly lower, with only 32.7% of carriage strains expressing MspA, dropping significantly to 13.7% for invasive isolates. Like NalP, this indicates a differential requirement for MspA expression in MenB and MenY strains. MspA-expressing *E. coli* showed significantly increased adherence *in vitro* to human cells compared to non-expressing cells [11], however in a separate study, the adhesion of *N. meningitidis* to various human epithelial and monocyte cell lines was unaffected by *mspA* mutation in a MenB strain [15]. Furthermore, inactivation of *mspA* did not influence other phenotypes including host cell invasion, utilization of hemoglobin and hemin as iron sources, hemagglutination nor serum resistance [15]. Thus, further studies are required to determine the function of MspA, and to explain the consequences of differential rates of MspA expression amongst serogroups. This differential expression may be important in meningococcal disease as MenY strains are associated with a predilection for infecting older age groups and causing pneumonia, whilst MenB disease (septicemia and meningitis) is more prevalent in infants and toddlers [33]. Finally, our data also shows that although *mspA* is highly prevalent in invasive strains, the development of clinical disease is not dependent *per se* on MspA expression since strains lacking the gene or harboring mutations that would prevent MspA expression (irrespective of phase ON/OFF status) can be found in collections of invasive strains. As with *nalP*, this may indicate redundancy and the presence of another protein which could replace the functionality of MspA in these strains.

In summary, two phase-variably expressed meningococcal autotransporters, NalP and MspA, are highly prevalent in both carriage and disease isolates. In some cases, differences in the proportions of strains in ON/OFF expression states were apparent between carriage and invasive isolate collections, and between serogroups, indicating differential requirements for MspA and NalP expression in different niches and in different meningococcal lineages. This analysis is a step towards understanding the potential contributions of phase variable loci to meningococcal carriage and disease, and also shows the utility of having a freely-available source of large numbers of bacterial genomes representative of a particular source or phenotype.

## Supporting Information

Figure S1 **Distribution of** ***nalP*** **tract lengths in meningococcal isolates.** (A) serogroup B strains only (B) serogroup Y strains only (C) all other serogroups. Black bars, carriage; grey bars, invasive. An ON PV state is produced by 7, 10 or 13Cs for *nalP*.
(PPT)

Figure S2 **Distribution of mspA tract lengths in meningococcal isolates.** (A) serogroup B strains only (B) serogroup Y strains only (C) all other serogroups. Black bars, carriage; grey bars, invasive. An ON PV state is produced by 6, 9 or 12Cs for mspA.
(PPT)

Table S1 **Invasive isolates in the Meningitis Research Foundation Meningococcus Genome Library database (containing the genomic DNA sequences of all disease isolates for 2010–11 in England, Wales and Northern Ireland) which lacked** ***mspA.***
(DOCX)

Table S2 **Invasive isolates in the Meningitis Research Foundation Meningococcus Genome Library database (containing the genomic DNA sequences of all disease isolates for 2010–11 in England, Wales and Northern Ireland) which lacked** ***nalP.***
(DOCX)
