# Supplementary figures and images for: Prevalence and Phase Variable Expression Status of Two Autotransporters, NalP and MspA, in Carriage and Disease Isolates of Neisseria meningitidis
Source: PLoS One. 2013 Jul 25;8(7):e69746. doi: 10.1371/journal.pone.0069746 (PMC3723659; doi:10.1371/journal.pone.0069746)

## Slide 1
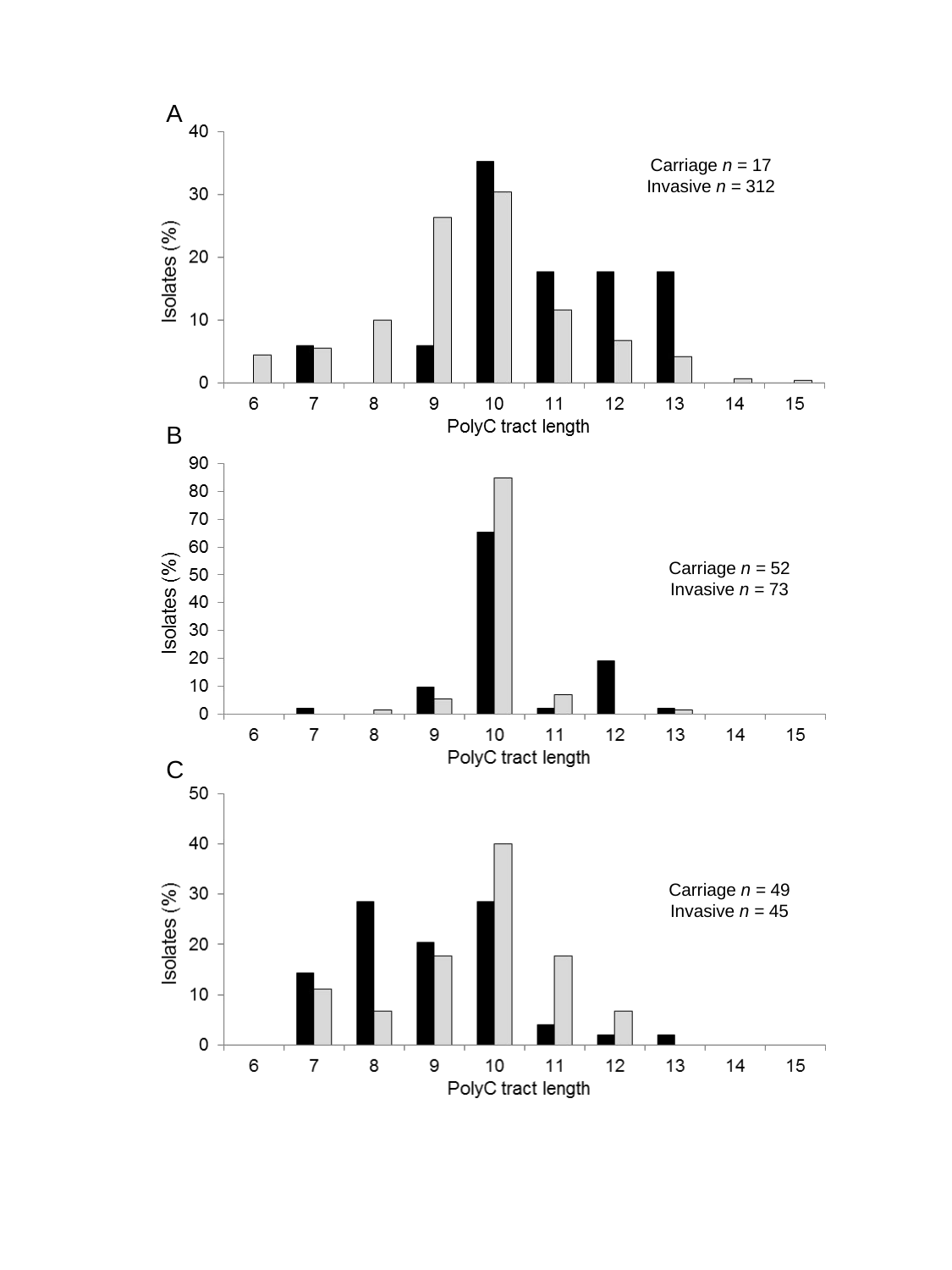

A
Carriage n = 17
Invasive n = 312
B
Carriage n = 52
Invasive n = 73
C
Carriage n = 49
Invasive n = 45

Supplement: Figure S1 — Distribution of nalP tract lengths in meningococcal isolates. (A) serogroup B strains only (B) serogroup Y strains only (C) all other serogroups. Black bars, carriage; grey bars, invasive. An ON PV state is produced by 7, 10 or 13Cs for nalP. (PPT) [file pone.0069746.s001.ppt]

## Slide 1
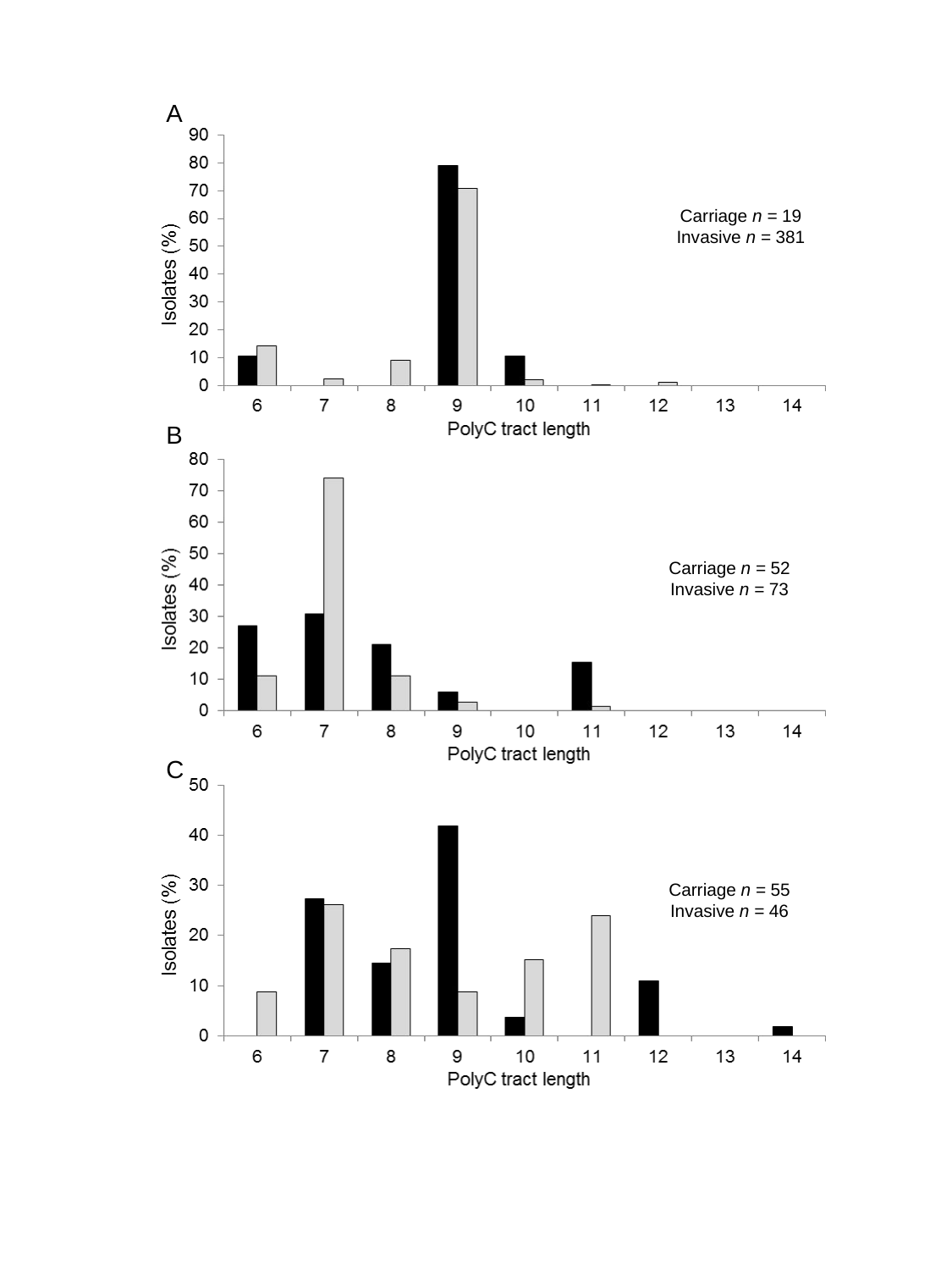

A
Carriage n = 19
Invasive n = 381
B
Carriage n = 52
Invasive n = 73
C
Carriage n = 55
Invasive n = 46

Supplement: Figure S2 — Distribution of mspA tract lengths in meningococcal isolates. (A) serogroup B strains only (B) serogroup Y strains only (C) all other serogroups. Black bars, carriage; grey bars, invasive. An ON PV state is produced by 6, 9 or 12Cs for mspA. (PPT) [file pone.0069746.s002.ppt]
