# Supplementary material for: Prevalence and Phase Variable Expression Status of Two Autotransporters, NalP and MspA, in Carriage and Disease Isolates of Neisseria meningitidis
Source: PLoS One. 2013 Jul 25;8(7):e69746. doi: 10.1371/journal.pone.0069746 (PMC3723659; doi:10.1371/journal.pone.0069746)
Supplement: Table S1 — Invasive isolates in the Meningitis Research Foundation Meningococcus Genome Library database (containing the genomic DNA sequences of all disease isolates for 2010–11 in England, Wales and Northern Ireland) which lacked mspA. (DOCX) [file pone.0069746.s003.docx]

| **Isolate** | **Serogroup** | **ST** | **Clonal complex** | **PorA VR1** | **PorA VR2** | **FetA VR** |
| --- | --- | --- | --- | --- | --- | --- |
| M10_240569 | B | 18 | ST-18 complex | 22 | 14 | F1-25 |
| M10_240683 | B | 18 | ST-18 complex | 22 | 9 | F5-5 |
| M11_240133 | B | 18 | ST-18 complex | 22 | 14 | F3-6 |
| M11_240045 | B | 858 | ST-18 complex | 5-1 | 2-2 | F5-16 |
| M11_240084 | B | 4997 | ST-18 complex | 22 | 14 | F3-6 |
| M11_240262 | A | 4789 | ST-5 complex/subgroup III | 20 | 9 | F3-1 |
| M11_240437 | Y | 784 | ST-92 complex | 5-1 | 10-4 | F4-3 |
| M11_240249 | B | 8944 | Not assigned | 21 | 16 | F1-7 |
| M11_240402 | B | 9825 | Not assigned | 21 | 16 | F3-7 |
| M11_240233 | NG | 9835 | ST-226 complex | 18-1 | 3 | F1-23 |
| M11_240287 | B | 9846 | ST-865 complex | 5-2 | 10-1 | F4-1 |
