# Supplementary material for: Prevalence and Phase Variable Expression Status of Two Autotransporters, NalP and MspA, in Carriage and Disease Isolates of Neisseria meningitidis
Source: PLoS One. 2013 Jul 25;8(7):e69746. doi: 10.1371/journal.pone.0069746 (PMC3723659; doi:10.1371/journal.pone.0069746)
Supplement: Table S2 — Invasive isolates in the Meningitis Research Foundation Meningococcus Genome Library database (containing the genomic DNA sequences of all disease isolates for 2010–11 in England, Wales and Northern Ireland) which lacked nalP. (DOCX) [file pone.0069746.s004.docx]

| **Isolate** | **Serogroup** | **ST** | **Clonal complex** | **PorA VR1** | **PorA VR2** | **FetA VR** | **NalP deletion class*^a^*** |
| --- | --- | --- | --- | --- | --- | --- | --- |
| M11_240094 | C | 5133 | ST-103 complex | deleted | deleted | F3-9 | NK |
| M11_240376 | B | 269 | ST-269 complex | 17-1 | 23 | F5-1 | Δ3 |
| M11_240275 | B | 269 | ST-269 complex | 19-1 | 15 | F5-1 | NK |
| M11_240077 | B | 269 | ST-269 complex | 19-1 | 15-11 | F1-7 | Δ3 |
| M10_240670 | B | 269 | ST-269 complex | 19-1 | 15-11 | F3-7 | NK |
| M10_240779 | B | 269 | ST-269 complex | 19-1 | 15-11 | F3-7 | NK |
| M11_240132 | B | 269 | ST-269 complex | 19-1 | 15-11 | F3-7 | Δ3 |
| M10_240474 | B | 269 | ST-269 complex | 19-1 | 15-11 | F3-9 | Δ3 |
| M11_240251 | B | 269 | ST-269 complex | 19-1 | 15-11 | F4-1 | NK |
| M10_240527 | B | 269 | ST-269 complex | 19-1 | 15-11 | F5-1 | NK |
| M10_240682 | B | 269 | ST-269 complex | 19-1 | 15-11 | F5-1 | NK |
| M10_240704 | B | 269 | ST-269 complex | 19-1 | 15-11 | F5-1 | NK |
| M10_240743 | B | 269 | ST-269 complex | 19-1 | 15-11 | F5-1 | NK |
| M11_240070 | B | 269 | ST-269 complex | 19-1 | 15-11 | F5-1 | NK |
| M11_240226 | B | 269 | ST-269 complex | 19-1 | 15-11 | F5-1 | NK |
| M11_240317 | B | 269 | ST-269 complex | 19-1 | 15-11 | F5-1 | NK |
| M11_240368 | B | 269 | ST-269 complex | 19-1 | 15-11 | F5-1 | NK |
| M11_240375 | B | 269 | ST-269 complex | 19-1 | 15-11 | F5-1 | NK |
| M11_240010 | B | 269 | ST-269 complex | 19-1 | 26 | F5-1 | NK |
| M10_240612 | B | 269 | ST-269 complex | 22 | 14 | F5-1 | NK |
| M11_240409 | B | 269 | ST-269 complex | 22 | 14 | F5-1 | Δ3 |
| M10_240515 | B | 269 | ST-269 complex | 5-1 | 2-2 | F5-1 | Δ3 |
| M11_240030 | B | 269 | ST-269 complex | 5-1 | 2-2 | F5-1 | NK |
| M10_240645 | B | 283 | ST-269 complex | 19-1 | 15-11 | F5-1 | NK |
| M10_240789 | B | 283 | ST-269 complex | 19-1 | 30 | F5-1 | NK |
| M11_240422 | B | 467 | ST-269 complex | 12-1 | 13-7 | F1-7 | NK |
| M10_240631 | C | 467 | ST-269 complex | 19-1 | 15-11 | F1-7 | NK |
| M10_240720 | B | 479 | ST-269 complex | 17 | 13-1 | F5-97 | NK |
| M10_240709 | B | 479 | ST-269 complex | 19 | 13-1 | F5-1 | NK |
| M11_240151 | B | 479 | ST-269 complex | 19 | 13-1 | F5-97 | NK |
| M10_240766 | B | 479 | ST-269 complex | 21 | 16 | F5-1 | NK |
| M11_240316 | B | 479 | ST-269 complex | 21 | 16 | F5-1 | NK |
| M10_240487 | B | 1049 | ST-269 complex | 19-1 | 15-11 | F5-1 | NK |
| M10_240729 | B | 1049 | ST-269 complex | 19-1 | 15-11 | F5-1 | Δ3 |
| M11_240025 | B | 1049 | ST-269 complex | 19-1 | 15-11 | F5-1 | NK |
| M11_240026 | B | 1049 | ST-269 complex | 19-1 | 15-11 | F5-1 | NK |
| M11_240180 | B | 1049 | ST-269 complex | 19-1 | 15-11 | F5-1 | NK |
| M11_240185 | B | 1049 | ST-269 complex | 19-1 | 15-11 | F5-1 | NK |
| M10_240808 | B | 1092 | ST-269 complex | 7-2 | 30 | F5-1 | NK |
| M10_240770 | B | 1195 | ST-269 complex | 22 | 9 | F5-1 | NK |
| M10_240708 | B | 1195 | ST-269 complex | 7-2 | 4 | F5-1 | NK |
| M11_240086 | B | 1774 | ST-269 complex | 17 | 16-3 | F5-1 | NK |
| M10_240768 | B | 2873 | ST-269 complex | 19 | 15-21 | F5-1 | NK |
| M11_240150 | B | 7939 | ST-269 complex | 5-1 | 2-2 | F5-1 | NK |
| M11_240388 | B | 9823 | ST-269 complex | 22 | 14 | F5-1 | NK |
| M11_240265 | B | 9836 | ST-269 complex | 19-1 | 15-11 | F5-1 | NK |
| M10_240728 | B | 9840 | ST-269 complex | 18-1 | 3 | F5-1 | NK |
| M11_240044 | B | 9843 | ST-269 complex | 22 | 9 | F5-1 | NK |
| M11_240280 | B | 278 | ST-35 complex | 7-2 | 13 | F4-1 | NK |
| M10_240809 | B | 41 | ST-41/44 complex/Lineage 3 | 7-2 | 4 | F1-5 | NK |
| M10_240548 | B | 340 | ST-41/44 complex/Lineage 3 | 7-2 | 4 | F1-5 | NK |
| M11_240125 | B | 3818 | ST-41/44 complex/Lineage 3 | 7-2 | 4 | F1-47 | NK |
| M10_240489 | B | 461 | ST-461 complex | 19-2 | 13-1 | F3-9 | Δ2 |
| M10_240716 | B | 461 | ST-461 complex | 19-2 | 13-1 | F3-9 | Δ2 |
| M11_240052 | B | 461 | ST-461 complex | 19-2 | 13-1 | F5-5 | Δ2 |
| M11_240083 | B | 461 | ST-461 complex | 19-2 | 13-1 | F5-5 | Δ2 |
| M11_240206 | B | 461 | ST-461 complex | 19-2 | 13-1 | F5-5 | Δ2 |
| M10_240591 | B | 461 | ST-461 complex | 7-1 | 4-1 | F3-9 | Δ2 |
| M11_240042 | B | 1946 | ST-461 complex | 19-2 | 13 | F3-9 | NK |
| M11_240001 | B | 1946 | ST-461 complex | 19-2 | 13-1 | F3-9 | Δ2 |
| M11_240214 | B | 1946 | ST-461 complex | 19-2 | 13-1 | F3-9 | NK |
| M11_240014 | B | 9891 | ST-461 complex | 19-2 | 13-7 | F3-9 | Δ2 |
| M11_240002 | B | 4954 | Not assigned | 19 | 15-1 | F5-2 | NK |

*^a^* NK, not known
